# Good News

**Published:** 1885-02

**Authors:** 


### GOOD NEWS.

Our good friend, Dr. Wells, writes us he is preparing a series of papers on the differentiation of remedies. The first will be on Aconite, Belladonna, and Bryonia. These papers will certainly be invaluable to the homœopathic prescriber, and with the lectures of Professor Kent, make this journal of yet greater usefulness.

